# Supervised Machine Learning Approach to Identify Early Predictors of Poor Outcome in Patients with COVID-19 Presenting to a Large Quaternary Care Hospital in New York City

**DOI:** 10.3390/jcm10163523

**Published:** 2021-08-11

**Authors:** Jason Zucker, Angela Gomez-Simmonds, Lawrence J. Purpura, Sherif Shoucri, Elijah LaSota, Nicholas E. Morley, Brit W. Sovic, Marvin A. Castellon, Deborah A. Theodore, Logan L. Bartram, Benjamin A. Miko, Matthew L. Scherer, Kathrine A. Meyers, William C. Turner, Maureen Kelly, Martina Pavlicova, Cale N. Basaraba, Matthew R. Baldwin, Daniel Brodie, Kristin M. Burkart, Joan Bathon, Anne-Catrin Uhlemann, Michael T. Yin, Delivette Castor, Magdalena E. Sobieszczyk

**Affiliations:** 1 Division of Infectious Diseases, Columbia University Irving Medical Center, New York, NY 10032, USA; ag2845@cumc.columbia.edu (A.G.-S.); lp2745@cumc.columbia.edu (L.J.P.); sms2319@cumc.columbia.edu (S.S.); bws2125@cumc.columbia.edu (B.W.S.); mc4846@cumc.columbia.edu (M.A.C.); dat2132@cumc.columbia.edu (D.A.T.); bm2266@cumc.columbia.edu (B.A.M.); mls97@cumc.columbia.edu (M.L.S.); au2110@cumc.columbia.edu (A.-C.U.); mty4@cumc.columbia.edu (M.T.Y.); dc2022@cumc.columbia.edu (D.C.); mes52@cumc.columbia.edu (M.E.S.); 2 Tulane University School of Medicine, Tulane Medical Center, New Orleans, LA 70112, USA; elijah.lasota@gmail.com; 3 Columbia University Vagelos College of Physicians and Surgeons, Columbia University Irving Medical Center, New York, NY 10032, USA; nem2147@cumc.columbia.edu; 4 Division of Infectious Diseases, Icahn School of Medicine at Mount Sinai, New York, NY 10029, USA; logan.bartram@mssm.edu; 5 Aaron Diamond AIDS Research Center, Vagelos College of Physicians and Surgeons, New York, NY 10032, USA; kam2157@cumc.columbia.edu; 6 General Internal Medicine, Columbia University Irving Medical Center, New York, NY 10032, USA; wt62@cumc.columbia.edu (W.C.T.); mk2748@cumc.columbia.edu (M.K.); 7 Mailman School of Public Health, Columbia University Irving Medical Center, New York, NY 10032, USA; pavlicov@gmail.com (M.P.); cale.basaraba@nyspi.columbia.edu (C.N.B.); 8 Division of Pulmonology, Columbia University Irving Medical Center, New York, NY 10032, USA; mrb45@cumc.columbia.edu (M.R.B.); hdb5@cumc.columbia.edu (D.B.); kb2319@cumc.columbia.edu (K.M.B.); 9 Division of Rheumatology, Columbia University Irving Medical Center, New York, NY 10032, USA; jmb2311@cumc.columbia.edu

**Keywords:** COVID, outcomes, machine learning

## Abstract

Background: The progression of clinical manifestations in patients with coronavirus disease 2019 (COVID-19) highlights the need to account for symptom duration at the time of hospital presentation in decision-making algorithms. Methods: We performed a nested case–control analysis of 4103 adult patients with COVID-19 and at least 28 days of follow-up who presented to a New York City medical center. Multivariable logistic regression and classification and regression tree (CART) analysis were used to identify predictors of poor outcome. Results: Patients presenting to the hospital earlier in their disease course were older, had more comorbidities, and a greater proportion decompensated (<4 days, 41%; 4–8 days, 31%; >8 days, 26%). The first recorded oxygen delivery method was the most important predictor of decompensation overall in CART analysis. In patients with symptoms for <4, 4–8, and >8 days, requiring at least non-rebreather, age ≥ 63 years, and neutrophil/lymphocyte ratio ≥ 5.1; requiring at least non-rebreather, IL-6 ≥ 24.7 pg/mL, and D-dimer ≥ 2.4 µg/mL; and IL-6 ≥ 64.3 pg/mL, requiring non-rebreather, and CRP ≥ 152.5 mg/mL in predictive models were independently associated with poor outcome, respectively. Conclusion: Symptom duration in tandem with initial clinical and laboratory markers can be used to identify patients with COVID-19 at increased risk for poor outcomes.

## 1. Introduction

Severe acute respiratory syndrome coronavirus 2 (SARS-CoV-2) has caused a global pandemic with over 30 million cases and 500,000 deaths in the United States alone [1]. In the spring of 2020, New York City was the first epicenter of the national outbreak as hospitals became overrun and hundreds of patients died from coronavirus disease 2019 (COVID-19) each day [2]. Since then, improved treatments have led to better outcomes in patients with COVID-19 [3]. However, while most cases are mild, mortality rates among hospitalized patients remain elevated [4,5].

Despite the rapid deployment of effective vaccines, rates of new cases of COVID-19, fueled by more transmissible variants, have plateaued [1,6,7]. Risk factors for severe disease include age, race/ethnicity, and underlying comorbidities, in addition to the degree of hypoxemia on presentation and a range of abnormal laboratory parameters [8,9,10]. For patients that develop severe or critical disease, complications include acute respiratory distress syndrome (ARDS), cytokine storm syndrome, and multiorgan dysfunction [11,12]. The pace of disease progression is variable but previous studies indicate that clinical deterioration typically occurs approximately five to seven days after symptom onset [12]. Predicting exactly which patients are most likely to require mechanical ventilation or die has been an area of active research [8,9,10,11,12,13]. To this end, multiple predictive scoring systems have been developed to assist clinicians in identifying such patients [14,15,16,17,18]. While most studies have relied on clinical and laboratory data upon hospital admission, to our knowledge none have analyzed these characteristics based on the duration of patients’ COVID-19 symptoms.

Given the delay between patients’ first symptoms and subsequent clinical decompensation, we hypothesized that the predictive value of clinical and laboratory parameters varies according to the duration of illness at the time of hospital presentation. We utilized a supervised machine learning approach to identify clinical comorbidities, initial vital signs, and laboratory markers associated with poor outcome in patients with COVID-19. Taking into account the duration of symptoms, we aimed to provide clinicians with practical algorithms for using clinical and laboratory markers to identify patients who may benefit from close monitoring and anticipatory management. 

## 2. Materials and Methods

**Figure 1:**
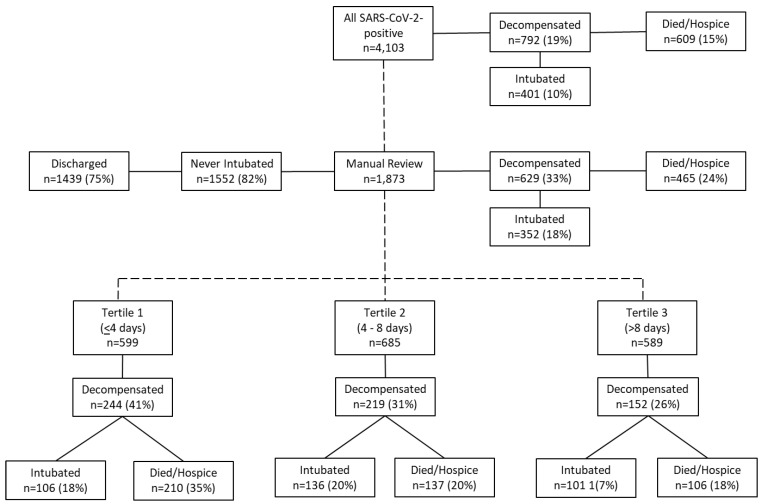
Flow chart of nested case–control study design.

We included all adult patients (≥18 years old) testing positive for SARS-CoV-2 who presented to a quaternary care medical center in Northern Manhattan between 1 March 2020 and 15 April 2020 and were evaluated in the emergency department (ED) (*n* = 4103) (Figure 1). Infection was confirmed by detection of SARS-CoV-2 by real-time reverse transcriptase polymerase chain reaction (RT-PCR) testing of nasopharyngeal and/or oropharyngeal swab specimens [19]. All patients had at least 28 days of follow-up after testing SARS-CoV-2-positive to allow adequate time to observe disease outcome. The institutional review board at Columbia University Irving Medical Center approved this under an expedited review (protocol number AAAS9622).

Data were extracted electronically from the electronic medical record (EMR) and were augmented with manually abstracted data. Electronically extracted data included demographics, admission and discharge dates, and diagnosis codes used to identify patients with pre-existing medical conditions. We obtained initial vital signs, first recorded oxygen delivery method defined as 0 = none; 1 = nasal cannula; 2 = non-rebreather, allowing for higher oxygen concentrations; and 3 = non-invasive ventilation, including high-flow nasal cannula, continuous, or bilevel positive airway pressure (CPAP and BIPAP, respectively). Patients who had mechanical ventilation as an initial recorded oxygen intervention were excluded, since mechanical ventilation was included as part of the outcome (N = 57). Basic laboratory results (complete blood count, basic metabolic panel, hepatic panel, prothrombin time, partial thromboplastin time, international normalized ratio), inflammatory markers, and other laboratory parameters were included in our institutional guidance for management of COVID-19 (erythrocyte sedimentation rate [ESR], C-reactive protein [CRP], lactate dehydrogenase [LDH], ferritin, d-dimer, procalcitonin, high-sensitivity troponin, and interleukin-6 [IL-6]). A subset of consecutive charts was manually reviewed starting with the first patient admitted with SARS-CoV-2 to our institution. Manually abstracted data, including the date of symptom onset and presenting symptoms, were entered into a REDCap database [20]. All data were merged using RStudio [21].

We conducted a nested case–control analysis to evaluate the association between initial vital signs and laboratory values, and the primary outcome. Cases were defined as patients who met the composite primary outcome of mechanical ventilation, death, or discharge to hospice with at least 28 days of follow-up. The remaining patients who survived to discharge or remained hospitalized but did not require intubation comprised the controls. Predictors included demographic, clinical, and laboratory data collected at the time of initial ED evaluation, and were typically obtained within 24 h of presentation. Investigational antivirals or immunomodulators targeting COVID-19 were inconsistently used during this time period and/or were not subsequently shown to improve outcomes in clinical studies and thus were not included as predictors in this analysis. Patients who underwent additional manual chart review and had a recorded date of symptom onset (*n* = 1873) were divided into tertiles based on number of days from symptom onset to hospital presentation (tertile 1, ≤4 days; tertile 2, >4–8 days; tertile 3, >8 days) for further analysis based on the duration of symptoms (Figure 1). 

All potential clinical and laboratory markers were described for the whole sample and for stratified tertiles described above. All continuous variables were non-normally distributed and tended to be outside of the normal range, and were described using medians and interquartile ranges (IQRs). Due to the data distribution and clinical context, we used classification and regression tree (CART) analysis, a non-parametric supervised machine learning approach, to determine the relative importance of clinical and laboratory predictors of poor outcome and to estimate clinically predictive threshold levels for continuous laboratory values [22]. For our full cohort, we used a training subset of 75% of patients to derive the predictive tree and a 25% partition to validate the prediction. Sample sizes in the analysis of symptom duration tertiles were too small to partition the data into training and validation subsets. Continuous and categorical variables were included. The full tree was gown using information gained measured by entropy. Regression tree analyses were conducted with complete set and missing values assigned using the “popular node” option to split nodes. In most instances, the “popular node” missing values reproduced the same tree as the complete data analysis, and thus were used. Pruning was determined as a function of cost-complexity and the estimated average misclassification rate in the leaves. In instances where the number of leaves were specified, these were revised to reduce overfitting (defined by having too few records in a leaf and only a single outcome). The confusion matrix and the area under the curve (AUC) were computed for all models. Additionally, unadjusted and multivariable logistic regression analyses were conducted for variables remaining in the pruned tree using the CART-informed threshold cut-off values to estimate the odds of poor COVID-19 outcome. Categorized clinical and laboratory markers that were significant in unadjusted logistic regressions were analyzed together in a multivariable logistic model. All statistical analyses and data visualization were performed in SAS^®^ software, version 9.4 (Cary, NC, USA), using the HPSPLIT procedure for the classification trees, and LOGISTIC procedure.

## 3. Results

### 3.1. Description of Clinical Characteristics and Laboratory Markers of the Cohort

During the study period, 4103 patients who tested positive for SARS-CoV-2 were evaluated in the ED. The median age was 63 years (IQR 49–75) and 54% were men. Patients were ethnically and racially diverse, as 36% of patients identified as Hispanic/Latino and 27% as Black (Table 1). The majority of patients reported fever (71%), cough (75%), and dyspnea (73%); other presenting symptoms were less common. The median body temperature at presentation was 99 °F (IQR 98.2–100.2 °F). Most did not require supplemental oxygen (59%) on presentation, although 17% were placed on non-rebreather and 3% required mechanical ventilation (excluded from inferential statistical analyses). There were 792 (19%) patients who met the primary outcome of intubation (*n* = 401, 10%) and/or death or discharge to hospice (*n* = 609, 15%) (Figure 1). 

### 3.2. Tertile Analysis Based on Duration of Symptom from Illness Onset to Hospital Presentation

There were 1873 (46%) patients for whom additional manual chart review was performed (Figure 1); compared to the overall cohort, a greater proportion of them were Hispanic and had underlying comorbidities, but presenting characteristics were otherwise similar. These patients were divided into tertiles defined by reported duration of symptoms at the time of presentation: ≤4 days (*n* = 599; tertile 1), 4–8 days (*n* = 685; tertile 2), or >8 days (*n* = 589; tertile 3).

Patients presenting earlier in their disease course were older with more comorbidities including hypertension, diabetes, and kidney disease than patients presenting in tertile 2 or tertile 3 (Table 1). Conversely, patients presenting later reported more symptoms including fever, cough, dyspnea, fatigue, myalgias, and diarrhea. The first reported method of oxygen delivery was similar across all three tertiles. Decompensations, however, were more common among patients in tertile 1: 244 (41%) decompensated compared to 219 (31%) and 152 (26%) of patients in tertiles 2 and 3, respectively (Figure 1). 

### 3.3. Classification and Regression Tree Analysis

**Figure 2:**
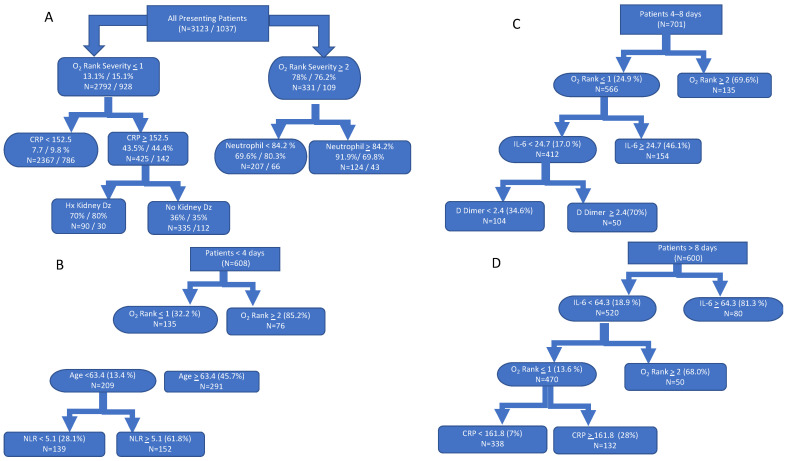
Classification and regression trees assessing predictors of poor outcome. Pruned classification trees that identified, ranked, and defined threshold cut points for predictors of decompensation in (**A**) all patients with COVID-19 presenting to the emergency department: first % represent prediction and second % represent validation; and in patients with a recorded date of symptom onset within (**B**) ≤4 days (tertile 1), (**C**) 4–8 days (tertile 2), and (**D**) >8 days (tertile 3) of hospital presentation. Decompensation was defined as a composite outcome of intubation, death, or discharge to hospice. Abbreviations and definitions: CRP, C-reactive protein; IL-6, interleukin-6; LDH, lactate dehydrogenase; NLR, neutrophil to lymphocyte ratio; OSR ≤ 1, nasal cannula; OSR ≥ 2, non-rebreather, non-invasive ventilation.

We used CART analysis to both rank predictors for the primary outcome and define clinically meaningful thresholds for determining risk (Figure 1). In the full prediction model including all patients regardless of symptom duration, the first recorded method of oxygen delivery was the most important predictor of poor outcome; 78% of patients requiring at least non-rebreather met the primary outcome, and 91.9% decompensated in the subset with a neutrophil percent ≥ 84%. In patients whose initial oxygen requirement did not exceed nasal cannula, 13% had a poor outcome, but this increased to 43.5% and 70% in patients with elevated CRP ≥ 152.5 μg/mL and kidney disease, respectively (Figure 2A). Findings were corroborated in the tree validation model using 25% partitioned data.

The pruned CART analyses stratified by duration of symptoms show different predictors, tree structure, and threshold cutoffs (Figure 2B–D). For patients in tertiles 1 and 2, the initial recorded method of oxygen delivery was the leading predictor of decompensation. The need for non-rebreather or greater oxygen requirement was significantly associated with decompensation. However, in tertile 1, age ≥ 63 years followed by NLR ≥ 5.1 were most useful for identifying high-risk patients, compared to IL-6 ≥ 24.7 pg/mL and D-dimer ≥ 2.4 µg/mL in tertile 2. In tertile 3, 81% of patients with IL-6 ≥ 64.3 pg/mL decompensated. Among those with IL-6 levels below the 64.3 pg/mL threshold, 68% of those who received non-rebreather decompensated (non-invasive ventilation was used by only one patient in this group). Elevated CRP (≥161.8 mg/L) predicted 28% decompensation among patients with lower supplemental oxygen requirements. All predictors identified in the pruned CART analysis were also independent predictors of decompensation in corresponding multivariable logistic regression models (Table S1). The dichotomized threshold cutoff values derived from the pruned CART analyses were strongly associated with poor outcomes in the multivariable logistic regression models (Table 2). For all models, the included predictors were found to be strongly and independently associated with poor outcomes. 

## 4. Discussion

Given the delay between patients’ first symptoms of COVID-19 and subsequent clinical decompensation, a better understanding of the initial clinical and laboratory parameters predictive of poor outcome are critical to guide clinical decision-making and management strategies. Here we evaluated the discriminatory ability of readily available clinical and laboratory parameters obtained at the time of hospital presentation to predict poor outcomes. Similar to previous reports, we found a broad range of predictors to be significantly associated with poor outcomes; however, we detected key differences in predictors including clinically meaningful laboratory thresholds that varied based on duration of symptoms at the time of hospital presentation. This corresponds to the evolution of signs and symptoms seen in patients with COVID-19 and likely reflects virologic progression and underlying host factors. 

Using CART analysis and subsequent multivariable logistic regression, we were able to incorporate interactions between variables to rank predictors in step-wise fashion and to estimate the likelihood for decompensation. Resulting models were simple and highlighted the predictive value of laboratory testing and other readily-available clinical information as part of the initial evaluation of patients with COVID-19. For example, in adjusted analysis, patients presenting within 4 days of symptom onset had a 7.4-, 4.8-, and 2.9-fold higher odds of decompensating if they required a non-rebreather or non-invasive ventilation, were ≥63 years, or had an NLR ≥5.1, respectively. Among patients presenting later in the disease course, 4–8 days, and >8 days after symptom onset, the adjusted odds of decompensating were 3.8 and 8.8 if they required a non-rebreather or non-invasive ventilation. While the initial type of supplemental oxygen was a highly important predictor of poor outcome overall and in most subgroup analyses, additional clinical and laboratory parameters varied in predicting decompensation but depended on patients’ duration of symptoms. IL-6 (pg/mL) thresholds of ≥24.7 and ≥64.3 were associated with 3.3- and 11.9-fold increased odds of decompensation in tertiles 2 and 3, respectively. D-dimer ≥ 2.4 (ug/mL) was associated with a 10% increased odds in tertile 2, and CRP ≥ 161.8 (mg/L) was associated with a 2-fold increased odds of decompensation in tertile 3. Finally, this approach generated clinically meaningful cutoffs for interpreting clinical and laboratory parameters, often differing substantially from usual clinical values. For example, while the established upper limit of the normal range of the IL-6 assay used at our institution is 1.8 pg/mL, a higher threshold value of 64.3 pg/mL identified high-risk patients from among those presenting after 8 days of symptoms. Cut-off values were supported by multivariable models but should be further validated in prospective studies.

We suspect that differences in CART models reflect evolving viral and host inflammatory responses accompanying COVID-19 disease progression [23]. Unsurprisingly, the need for higher levels of oxygen supplementation was the most predictive factor of poor outcome in this patient population, as pulmonary involvement is seen in most patients with COVID-19 presenting to the hospital. Based on our results, respiratory failure is an early indicator of decompensation, and the most important predictor in patients presenting within 8 days of symptom onset. Subsequently, mounting inflammatory responses and hypercoagulability may become more prominent, which in a subset of patients can result in development of cytokine storm, progressive ARDS, and other end-organ failure [24]. Correspondingly, elevated inflammatory markers such as IL-6, CRP, and D-dimer were found to be leading predictors of poor outcome in patients presenting later in their disease course. In patients presenting after 8 days of symptoms, elevated IL-6 > 64.3 pg/mL was the most important predictor of decompensation and may reflect development of inflammatory complications. Interestingly, symptom duration also appeared to vary across different patient demographics, e.g., younger patients and those with more medical comorbidities presenting earlier, which may reflect different disease phenotypes [25]. Further studies are needed to better understand the pathophysiologic changes underlying the temporal dynamics of laboratory and other clinical markers for disease progression in patients with COVID-19.

Our results expand on several previous studies that found specific initial laboratory parameters to be significant predictors of poor outcome in patients with COVID-19 in multivariable analyses. These studies similarly found lymphopenia, elevated troponin, renal and hepatic indices, low albumin, and elevated inflammatory markers, such as CRP, D-dimer, procalcitonin, and IL-6, to be independently associated with development of ARDS, need for intensive care unit admission or mechanical ventilation, and mortality [11,26,27,28,29,30,31,32,33]. Other studies identified parameters such as increased CD4/CD8 radio, which was not included among initial recommended laboratory tests at our medical center, to be significantly elevated in patients at increased risk for critical illness [34]. Wang et al. developed a laboratory-based model for predicting hospital mortality which included age, initial oxygen saturation, neutrophil and lymphocyte counts, CRP, D-dimer, AST, and glomerular filtration rate [35]. Two studies conducted in China and the UK used Lasso regression to derive predictive models for mortality and/or critical illness, which consisted of demographic and clinical variables as well as laboratory parameters such as NLR or elevated neutrophil count, CRP, LDH, creatinine, and albumin [14,15]. These variables were then used to build clinical risk scores for identifying high-risk patients. More recently, using multivariable logistic regression analysis, researchers developed risk scores for mechanical ventilation and in-hospital death in COVID-19 [16]. Oxygenation, CRP, and LDH levels, along with a history of diabetes mellitus were deemed significant risk factors for requiring mechanical ventilation; age, male sex, coronary artery disease, diabetes mellitus, chronic statin use, oxygenation, BMI, neutrophil-to-lymphocyte ratio, platelet count, and procalcitonin levels were associated with increased risk of in-hospital mortality. However, symptom duration was not directly accounted for in any these models.

Several limitations of our study need to be considered. This was a single-center study and thus our findings may not be applicable to hospitals in non-urban centers or with different patient populations or different protocols for testing and monitoring of immune markers. Because of the retrospective study design, we were limited to available clinical data that could be readily extracted from the EMR. The follow-up period was a minimum of 28 days, which may not have been sufficient for identifying all patients who would meet the primary outcome. However, the number of patients still hospitalized at the time the analysis was completed was relatively small. We assessed a large number of patients presenting to our hospital for evaluation, and while our complete data and imputed CART analysis were mostly consistent, there may be bias in the resulting trees based on either imputed or complete data. Our findings were corroborated with the validation sample for the larger sample set, but sample size limitations precluded validation of the tertile. In addition to reducing sample sizes, particularly in the tertile analyses, this may have introduced bias towards patients at increased risk for decompensation, as these patients may have been more likely to receive a complete evaluation. We also relied on self-reporting of symptom types and duration, which may have been subject to recall bias. However, substantial differences in outcomes between tertiles were detected, and we do not expect differential misclassification bias to have occurred. Finally, we included only clinical and laboratory data from the initial evaluation, and therefore were not able to include a broad range of dynamic factors and complications that may have contributed to outcomes. However, this was consistent with our goal of developing an early triage tool based on data collected at the time of initial hospital presentation.

## 5. Conclusions

Our findings support the continued use of simple, practical clinical decision-making tools for the initial management of patients with COVID-19, especially in hospital settings where rapid triage is needed, capacity is limited, and virtual assessments may be performed by consultants and other medical care providers. Our results indicate that initial laboratory markers play an important role in clinical algorithms for identifying patients at increased risk for having a poor outcome. Because predictive factors differ by duration of illness, the initial patient evaluation should attempt to determine the date of symptom onset. Additional studies are needed to validate our results in a prospective cohort and link them to more precise characterization of underlying pathophysiologic processes.

## Figures and Tables

**Table 1:** Demographic and clinical characteristics of patients diagnosed with COVID-19 overall and by duration from symptom onset.

	Overall Cohort			Tertiles: Days since Onset of Symptoms		
	All Patients (*n* = 4103)	Intubated/ Died (*n* = 792)	Survived without Intubation (*n* = 3311)	≤4 Days (*n* = 599)	4–8 Days (*n* = 685)	>8 Days (n = 589)
**Demographics, *n* (%)**						
**Sex**						
**Male**	2214 (53.96)	476 (60.10)	1738 (52.49)	341 (56.93)	391 (57.08)	333 (56.54)
**Female**	1889 (46.04)	316 (39.90)	1573 (47.51)	258 (43.07)	294 (42.92)	256 (43.46)
**Ethnicity**						
**Hispanic**	1483 (36.14)	387 (48.86)	1096 (33.10)	312 (52.09)	355 (51.82)	308 (52.29)
**Non-Hispanic**	1534 (37.39)	220 (27.78)	1314 (39.69)	162 (27.05)	176 (25.69)	138 (23.43)
**Unknown**	1086 (26.47)	185 (23.36)	901 (27.21)	125 (20.87)	154 (22.48)	143 (24.28)
**Race**						
**Black**	1088 (26.52)	152 (19.19)	936 (28.27)	139 (23.21)	151 (22.04)	103 (17.49)
**White**	1074 (26.18)	209 (26.39)	865 (26.13)	144 (24.04)	158 (23.07)	130 (22.07)
**Other**	1941 (47.31)	431 (54.42)	1510 (45.61)	316 (52.75)	376 (54.89)	356 (60.44)
**Age, median (IQR)**	63 (49, 75)	73 (62, 83)	60 (46, 72)	69 (55, 80)	62 (51, 75)	62 (47, 72)
**Symptoms, *n* (%)**						
**Fever**	1501 (70.64)	476 (70.73)	1025 (70.59)	360 (62.07)	520 (76.36)	445 (75.94)
**Chills**	688 (31.84)	168 (25.69)	500 (34.63)	134 (23.67)	239 (35.46)	234 (40.07)
**Fatigue**	723 (34.40)	183 (27.81)	540 (37.24)	145 (25.44)	271 (40.21)	252 (43.08)
**Dyspnea**	1560 (73.17)	556 (81.89)	1004 (69.10)	371 (63.75)	550 (80.65)	476 (81.23)
**Chest Pain**	342 (16.19)	74 (11.23)	268 (18.44)	76 (13.31)	109 (16.1)	123 (21.03)
**Cough**	1603 (75.26)	483 (71.88)	1120 (76.82)	365 (62.93)	566 (82.99)	497 (84.67)
**Nausea**	418 (19.86)	78 (11.87)	340 (23.48)	92 (16.2)	136 (20.18)	155 (26.41)
**Diarrhea**	503 (23.92)	113 (17.28)	390 (26.92)	66 (11.64)	195 (28.89)	197 (33.62)
**Myalgia**	571 (27.26)	110 (16.95)	461 (31.88)	95 (16.99)	208 (30.81)	217 (37.03)
**Comorbidities, *n* (%)**						
**Diabetes**	990 (24.13)	355 (44.82)	635 (19.18)	266 (44.41)	264 (38.54)	195 (33.11)
**Hypertension**	1549 (37.75)	547 (69.07)	1002 (30.26)	412 (68.78)	386 (56.35)	326 (55.35)
**Pulmonary Disease**	470 (11.89)	153 (19.32)	317 (9.57)	119 (19.87)	142 (20.73)	102 (17.32)
**Kidney Disease**	488 (11.89)	245 (30.93)	243 (7.34)	144 (24.04)	136 (19.85)	91 (15.45)
**Liver Disease**	139 (3.39)			36 (6.01)	23 (3.36)	35 (5.94)
**Use of ACE Inhibitor**	118 (15.51)	35 (15.35)	83 (15.57)	40 (14.71)	43 (20.87)	22 (14.47)
**Vital signs and laboratory parameters, median (IQR)**						
**Body Mass Index**	28.14 (24.5, 32.8)	27.1 (23.8, 31.69)	28.52 (25, 33.2)	27.38 (23.44, 31.67)	28.51 (25.3, 33.37)	29.03 (25.63, 33.89)
**OSR, *n* (%)**						
**OSR = 0**	1264 (58.65)	304 (40.05)	960 (68.77)	329 (60.37)	373 (57.12)	330 (60.33)
**OSR = 1**	508 (23.57)	170 (22.40)	338 (24.21)	117 (21.47)	161 (24.66)	150 (27.42)
**OSR = 2**	377 (17.49)	280 (36.89)	97 (6.95)	97 (17.8)	117 (17.92)	66 (12.07)
**OSR = 3**	6 (0.28)	5 (0.66)	1 (0.07)	2 (0.37)	2 (0.31)	1 (0.18)
**Initial** **Temperature** **(°F)**	99.0	98.9	99	98.8	99.1	99.1
	(98.2, 100.2)	(98.1, 100.2)	(98.2, 100.0)	(98.2, 100.3)	(98.2, 100.5)	(98.2, 100.4)
**WBC Count** **(×10^3^/µL)**	7.29	8.45	6.90	7.08	7.05	7.61
	(5.39, 9.92)	(5.98, 11.87)	(5.25, 9.14)	(5.3, 9.88)	(5.35, 9.75)	(5.75, 9.94)
**Neutrophil %**	76.9	81.7	74.2	75.3	76.7	77.8
	(68.3, 83.8)	(74.95, 86.9)	(66, 81.3)	(66.3, 83.5)	(68.6, 83.3)	(70.1, 84)
**Lymphocyte %**	14	10.45	16.2	14.2	14.35	13.4
	(9, 21)	(6.5, 16.1)	(10.5, 22.8)	(8.6, 21.3)	(9.3, 21.3)	(9.1, 20.5)
**NLR**	5.49	7.8	4.58	5.34	5.30	5.87
	(3.27, 9.22)	(4.7, 13.38)	(2.88, 7.66)	(3.15, 9.49)	(3.2, 8.84)	(3.46, 9.09)
**Hemoglobin (g/dL)**	13.1	13.1	13.1	13	13.4	13.4
	(11.7, 14.5)	(11.4, 14.5)	(11.8, 14.5)	(11.2, 14.5)	(12, 14.7)	(12, 14.6)
**Platelet Count** **(×10^3^/µL)**	199	191.5	202	185	195	215
	(153, 257.5)	(146, 260)	(156, 256)	(142, 242)	(154, 251)	(168, 267)
**Creatinine (mg/dL)**	1.06	1.3	1	1.21	1.05	0.99
	(0.8, 1.61)	(0.91, 2.24)	(0.76, 1.4)	(0.85, 1.98)	(0.81, 1.52)	(0.76, 1.33)
**Albumin (g/dL)**	3.8 (3.4, 4.1)	3.6 (3.2, 3.8)	3.9 (3.5, 4.2)	3.8 (3.4, 4.2)	3.8 (3.5, 4.1)	3.8 (3.4, 4.1)
**AST (U/L)**	42 (27, 68)	55 (35, 87)	37 (25, 59)	38 (24, 65)	42 (30, 68)	46 (30, 69)
**ALT (U/L)**	28 (18, 48)	30 (20, 53)	27 (18, 45)	25 (17, 43)	29 (19, 48)	32 (21, 53)
**ESR (mm/hr)**	70 (48, 96)	75 (55, 101)	67 (45, 91)	67 (43, 94)	69 (48, 95)	72 (54, 98)
**CRP (mg/L)**	113.8	167.92	91.9	99.33	113.82	133.64
	(56.47, 198.64)	(99.33, 260.89)	(39.54, 160.97)	(35.95, 178.2)	(59.7, 205.1)	(72.25, 204.46)
**LDH (U/L)**	407	516	363	361	411	422.5
	(297, 559)	(377, 708)	(273, 479)	(258, 523)	(308, 575)	(326, 552)
**Ferritin (ng/mL)**	686.5	852.6	591.1	604.5	667.8	793.65
	(336.8, 1258)	(453.4, 1546)	(282.3, 1115)	(277.7, 1262)	(356.8, 1239)	(407, 1395)
**D-Dimer** **(µg/mL)**	1.42	2.42	1.15	1.47	1.24	1.34
	(0.81, 3.17)	(1.23, 6.27)	(0.68, 2.23)	(0.83, 3.22)	(0.72, 2.59)	(0.78, 2.86)
**Procalcitonin (ng/mL)**	0.23	0.45	0.16	0.26	0.2	0.2
	(0.11, 0.59)	(0.20, 1.27)	(0.09, 0.37)	(0.12, 0.9)	(0.11, 0.46)	(0.1, 0.49)
**IL-6** **(pg/mL)**	19.5	45.08	12.05	19.2	16	19.7
	(6, 49)	(18, 92.48)	(5, 29.7)	(5, 48.38)	(5.78, 42.95)	(7.23, 49.3)
**Troponin (ng/L)**	16 (8, 42)	31(15, 76)	12 (6, 25)	26 (11, 63)	13 (7, 30)	11 (6, 23)

Abbreviations: OSR, oxygen severity rank; WBC, white blood cell; NLR, neutrophil to lymphocyte ratio; AST, aspartate aminotransferase; ALT, alanine aminotransferase; PT, prothrombin time; PTT, partial thromboplastin time; CRP, C-reactive protein; ESR, erythrocyte sedimentation rate; LDH, lactate dehydrogenase; IL-6, interleukin-6.

**Table 2:** Multivariable logistic regression model of predictive variables identified through classification tree analysis.

	Overall Cohort		Tertiles Defined by Symptom Duration					
	All Patients (*n* = 4103)		≤4 Days (*n* = 599)		4–8 Days (*n* = 685)		>8 Days (*n* = 589)	
Predictors	Crude	Adjusted	Crude	Adjusted	Crude	Adjusted	Crude	Adjusted
	OR (95%CI)	OR (95%CI)	OR (95%CI)	OR (95%CI)	OR (95%CI)	OR (95%CI)	OR (95%CI)	OR (95%CI)
**OSR ≤ 1** **(Nasal cannula)**	Ref							
**OSR ≥ 2** **(Non-rebreather, non-invasive ventilation)**	8.0	5.5	9.6	7.4	5.4	3.8	11.2	8.8
	(6.18–10.25)	(4.15–7.32)	(5.46–17.04)	(4.01–13.71)	(3.54–8.27)	(2.05–6.10)	(6.18, 20.21)	(4.12, 18.76)
**CRP ≥ 161.8 mg/L**	3.7	2.6					3.9	2.0
	(3.03, 4.44)	(2.04, 3.20)					(2.60–5.75)	(1.21–3.46)
**Neutrophil % ≥ 84.18**	3.4 (2.73, 4.13)	1.9 (1.46, 2.41)						
**Kidney disease**	5.7	2.7						
	(4.63, 6.90)	(2.10, 3.46)						
**IL-6 ≥ 24.7 pg/mL**					4.3	3.3		
					(2.91–6.30)	(2.15–5.01)		
**IL-6 ≥ 64.3 pg/mL**							16.1	11.9
							(8.6–30.1)	(6.0–23.63)
**Age ≥ 63 years**			4.8	4.8				
			(3.30–7.10)	(3.03–7.47)				
**NLR ≥ 5.1**			3.8	2.9				
			(2.66–5.48)	(1.93–4.42)				
**D-Dimer ≥ 2.4 µg/mL**					1.2	1.1		
					(1.12–1.24)	(1.06–1.19)		

Abbreviations: OSR, oxygen severity rank; NLR, neutrophil to lymphocyte ratio; CRP, C-reactive protein; LDH, lactate dehydrogenase; IL-6, interleukin-6.

## Data Availability

The data presented in this study are available upon request to the corresponding author.

## Supplementary Materials

The following are available online at https://www.mdpi.com/article/10.3390/jcm10163523/s1, Table S1: Multivariable logistic regression model for the association between baseline demographic, clinical, and laboratory markers and decompensation.
