# Supplementary material for: Supervised Machine Learning Approach to Identify Early Predictors of Poor Outcome in Patients with COVID-19 Presenting to a Large Quaternary Care Hospital in New York City
Source: J Clin Med. 2021 Aug 11;10(16):3523. doi: 10.3390/jcm10163523 (PMC8397083; doi:10.3390/jcm10163523)
Supplement: Supplementary file 1 [file jcm-10-03523-s001.zip › jcm-1286471-supplementary.pdf]

Table S1. Multivariable Logistic Regression Model for the Association between Baseline Demographic, Clinical and Laboratory Markers and Decompensation.

	Overall Cohort		Tertiles Defined by Symptom Duration					
	All Patients (n=4,103)		≤4 days (n=599)		4-8 days (n=685)		>8 days (n=589)	
Predictors	Crude OR (95%CI)	Adjusted OR (95%CI)	Crude OR (95%CI)	Adjusted OR (95%CI)	Crude OR (95%CI)	Adjusted OR (95%CI)	Crude OR (95%CI)	Adjusted OR (95%CI)
**Comorbidities**								
**Hypertension**	5.15** (4.35, 6.09)	0.92 (0.66, 1.3)	1.83* (1.27, 2.64)	1.13 (0.59, 2.16)	1.5* (1.08, 2.08)	0.89 (0.49, 1.62)	1.54* (1.06, 2.25)	0.74 (0.39, 1.4)
**Diabetes**	3.42** (2. 9, 4.04)	0.75 (0.55, 1.02)	1.2 (0.87, 1.67)		1.55* (1.12, 2.15)	0.55 (0.3, 1.01)	1.25 (0.85, 1.84)	
**Pulmonary Disease**	2.26** (1.83, 2.79)	1.01 (0.7, 1.46)	1.12 (0.74, 1.67)		0.80 (0.53, 1.2)		1.32 (0.83, 2.11)	
**Renal Disease**	5.66** (4.63, 6.90)	3.64** (2.49, 5.32)	1.42 (0.97, 2.07)		4.39** (2.96, 6.5)	6.9** (3.53, 13.5)	3.2** (2.01, 5.08)	2.76* (1.36, 5.61)
**Liver Disease**	1.26 (0.84, 1.89)		0.54 (0.26, 1.14)		0.74 (0.29, 1.91)		0.71 (0.3, 1.65)	
**Vital signs and laboratory parameters**								
**Body Mass Index**	1.00 (1.00, 1.00)		0.99 (0.97, 1.01)		1.00 (1.00, 1.00)		0.99 (0.97, 1.01)	
**ORS**	2.71** (2.4, 3.06)	1.68** (1.40, 2.02)	2.86** (2.23, 3.66)	1.44 (0.96, 2.15)	2.47** (1.99, 3.07)	1.93** (1.4, 2.66)	2.86** (2.18, 3.75)	1.87* (1.26, 2.78)
**Initial Temperature**	0.97 (0.92, 1.02)	1.04 (0.95, 1.13)	0.95 (0.86, 1.05)		0.93* (0.85, 1.03)	1.02 (0.87, 1.18)	1.03 (0.92, 1.16)	
**WBC Count**	1.09** (1.07, 1.11)	1.00 (0.97, 1.02)	1.10** (1.06, 1.14)	0.91* (0.83, 0.99)	1.12** (1.07, 1.17)	1.02 (0.98, 1.06)	1.07* (1.03, 1.11)	0.99 (0.95, 1.03)
**Neutrophil %**	1.07** (1.05, 1.08)		1.08** (1.06, 1.09)	1.02 (0.95, 1.09)	1.06 ** (1.04, 1.08)		1.06** (1.04, 1.09)	
**Lymphocyte %**	0.93** (0.92, 0.94)		0.92** (0.90, 0.94)	0.99 (0.90, 1.07)	0.92** (0.90, 0.95)		0.94** (0.91, 0.96)	
**NLR**	1.07** (1.05, 1.08)	1.00 (0.99, 1.01)	1.01 (0.99, 1.03)		1.10** (1.07, 1.14)	1.01 (0.97, 1.05)	1.13** (1.08, 1.17)	1.02 (0.97, 1.07)
**Hemoglobin**	0.95* (0.92, 0.99)	0.97 (0.90, 1.04)	0.96 (0.89, 1.02)		0.95 (0.88, 1.02)		0.99 (0.9, 1.08)	

**Platelet Count**	1.00<br>(1.00, 1.00)		1.00<br>(1.00, 1.00)		1.00<br>(1.00, 1.00)		1.00<br>(1.00, 1.00)	
**Creatinine**	1.09**<br>(1.05, 1.13)	0.94<br>(0.88, 1.00)	1.01<br>(0.95, 1.08)		1.05<br>(0.99, 1.12)		1.07<br>(0.97, 1.17)	
**Albumin**	0.3**<br>(0.25, 0.36)	0.67*<br>(0.48, 0.92)	0.26**<br>(0.19, 0.37)	0.51*<br>(0.28, 0.92)	0.35**<br>(0.25, 0.5)	0.90<br>(0.51, 1.6)	0.26**<br>(0.17, 0.39)	0.71<br>(0.37, 1.36)
**AST**	1.01**<br>(1.00, 1.01)	1.00<br>(1.00, 1.01)	1.00*<br>(1.00, 1.01)	1.00<br>(1.00, 1.00)	1.01*<br>(1.00, 1.01)	1.00<br>(1.00, 1.01)	1.01**<br>(1.00, 1.01)	1.01<br>(1.00, 1.02)
**ALT**	1.00*<br>(1.00, 1.00)	1.00<br>(0.99, 1.00)	1.00<br>(1.00, 1.00)		1.00<br>(1.00, 1.00)		1.00*<br>(1.00, 1.01)	1.00<br>(0.99, 1.01)
**ESR**	1.01**<br>(1.01, 1.01	0.99*<br>(0.99, 1.00)	1.01*<br>(1.00, 1.02)	0.99<br>(0.99, 1.00)	1.01*<br>(1.01, 1.02)	1.00<br>(0.99, 1.01)	1.01<br>(1.00, 1.01)	
**CRP**	1.01**<br>(1.01, 1.01)	1.00**<br>(1.00, 1.01)	1.01**<br>(1.01, 1.01)	1.00*<br>(1.00, 1.01)	1.01**<br>(1.01, 1.01)	1.00*<br>(1.00, 1.01)	1.01**<br>(1.01, 1.01)	1.00<br>(1.00, 1.01)
**LDH**	1.00**<br>(1.00, 1.00)	1.00**<br>(1.00, 1.00)	1.00**<br>(1.00, 1.01)	1.00*<br>(1.00, 1.01)	1.00**<br>(1.00, 1.00)	1.00*<br>(1.00, 1.00)	1.00**<br>(1.00, 1.00)	1.00<br>(1.00, 1.00)
**Ferritin**	1.00**<br>(1.00, 1.00)		1.00*<br>(1.00, 1.00)		1.00*<br>(1.00, 1.00)		1.00*<br>(1.00, 1.00)	
**D-Dimer**	1.00<br>(1.00, 1.00)		1.00<br>(1.00, 1.00)		1.18**<br>(1.12, 1.24)	1.08*<br>(1.01, 1.15)	1.00<br>(1.00, 1.00)	
**Procalcitonin**	1.02*<br>(1.01, 1.03)	1.01<br>(0.99, 1.02)	1.01<br>(1.00, 1.03)		1.06*<br>(1.01, 1.11)	1.00<br>(0.97, 1.02)	1.08*<br>(1.02, 1.14)	1.00<br>(0.97, 1.03)
**IL-6**	1.02**<br>(1.02, 1.02)	1.01**<br>(1.01, 1.02)	1.02**<br>(1.01, 1.03)	1.01<br>(1.00, 1.01)	1.02**<br>(1.01, 1.02)	1.01<br>(1.00, 1.01)	1.03**<br>(1.02, 1.04)	1.02**<br>(1.01, 1.03)
**Troponin**	1.01**<br>(1.00, 1.01)	1.00<br>(1.00, 1.00)	1.00**<br>(1.00, 1.01)	1.00<br>(1.00, 1.01)	1.01**<br>(1.00, 1.01)	1.00<br>(0.99, 1.00)	1.00<br>(1.00, 1.00)	

*Wald Chi Square p <0.05; **Wald Chi Square p <0.01

Abbreviations: ORS, oxygen rank severity; WBC, white blood cell; NLR, neutrophil to lymphocyte ratio; AST, aspartate aminotransferase; ALT, alanine aminotransferase; PT, prothrombin time; PTT, partial thromboplastin time; CRP, C-reactive protein; ESR, erythrocyte sedimentation rate; LDH, lactate dehydrogenase; IL-6, interleukin-6
